# Integrative metagenomic and metabolomic analyses reveal severity-specific signatures of gut microbiota in chronic kidney disease

**DOI:** 10.7150/thno.41725

**Published:** 2020-04-06

**Authors:** I-Wen Wu, Sheng-Siang Gao, Hsin-Cheng Chou, Huang-Yu Yang, Lun-Ching Chang, Yu-Lun Kuo, Michael Cong Vinh Dinh, Wen-Hung Chung, Chi-Wei Yang, Hsin-Chih Lai, Wen-Ping Hsieh, Shih-Chi Su

**Affiliations:** 1 Department of Nephrology, Chang Gung Memorial Hospital, Keelung, Taiwan; 2 College of Medicine, Chang Gung University, Taoyuan, Taiwan; 3 Institute of Statistics, National Tsing-Hua University, Hsinchu, Taiwan; 4 Kidney Research Center, Department of Nephrology, Chang Gung Memorial Hospital, Linkuo, Taiwan; 5 Department of Health Policy and Management, Johns Hopkins Bloomberg School of Public Health, Baltimore, Maryland, US; 6 Department of Mathematical Sciences, Florida Atlantic University, Florida, US; 7 Biotools, Co., Ltd, New Taipei City, Taiwan; 8 Department of Computer Science, Florida Atlantic University, Florida, US; 9 Whole-Genome Research Core Laboratory of Human Diseases, Chang Gung Memorial Hospital, Keelung, Taiwan; 10 Department of Medical Biotechnology and Laboratory Science, and Microbiota Research Center, College of Medicine, Chang Gung University, Taoyuan, Taiwan; 11 Central Research Laboratory, XiaMen Chang Gung Hospital, XiaMen, China; 12 Department of Laboratory Medicine, Chang Gung Memorial hospital, Linkuo, Taiwan

**Keywords:** Chronic kidney disease, Gut microbiome, Serum metabolites, Short-chain fatty acid, Uremic solute

## Abstract

Chronic kidney disease (CKD) is a serious healthcare dilemma, associated with specific changes in gut microbiota and circulating metabolome. Yet, the functional capacity of CKD microbiome and its intricate relationship with the host metabolism at different stages of disease are less understood. Methods: Here, shotgun sequencing of fecal samples and targeted metabolomics profiling of serum bile acids, short- and medium-chain fatty acids, and uremic solutes were performed in a cohort of CKD patients with different severities and non-CKD controls. Results: We identified that levels of 13 microbial species and 6 circulating metabolites were significantly altered across early to advanced stages or only in particular stage(s). Among these, Prevotella sp. 885 (decreased) was associated with urea excretion, while caproic acid (decreased) and p-cresyl sulfate (elevated) were positively and negatively correlated with the glomerular filtration rate, respectively. In addition, we identified gut microbial species linked to changes in circulating metabolites. Microbial genes related to secondary bile acid biosynthesis were differentially abundant at the early stage, while pathway modules related to lipid metabolism and lipopolysaccharide biosynthesis were enriched in the CKD microbiome at the advanced stage, suggesting that changes in microbial metabolism and host inflammation may contribute to renal health. Further, we identified metagenomic and metabolomic markers to discriminate cases of different severities from the controls, among which Bacteroides eggerthii individually was of particular value in early diagnosis. Conclusions: Our dual-omics data reveal the connections between intestinal microbes and circulating metabolites perturbed in CKD, which may be of etiological and diagnostic importance.

## Introduction

Chronic kidney disease (CKD) is a huge healthcare burden commonly associated with metabolic and cardiovascular comorbidities 1. Numerous risk factors, including age, gender, genetic inheritance, exposure to heavy metals, tobacco smoking, excessive alcohol use, virus infections, obesity, hypertension, and diabetes mellitus 2, have been demonstrated to be interrelated and imperative to estimate the prognosis of CKD and to affect its progression into end-stage renal disease (ESRD). Such complex etiology increases the clinical heterogeneity of the condition and urges for the identification of manipulable predisposing factors to CKD to hamper the disease progression to ESRD.

Emerging evidence has related CKD and ESRD to the microbial composition and function of the intestines 3-5. Although inconclusive, compositional alterations, with a significant enrichment of *Firmicutes*, *Actinobacteria*, and *Proteobacteria*, and a depletion in the abundance of *Bifidobacteria* and *Lactobacilli*, were observed in patients with ESRD as compared to normal controls 6-8. Notably, maladaptation of microflora to gut environmental changes can be detected at the early stage of this syndrome 9. Such gut dysbiosis, coupled with impaired intestinal barrier function dictate the overall inflammatory state in CKD by accommodating bacterial translocation and the presence of endotoxin and other noxious luminal products in the circulation 10. In addition to regulation of host immunity, gut bacteria function as an endocrine organ by producing diet-derived bioactive metabolites that could possess nephrotoxic or nephroprotective potential 11. In particular, some precursors of uremic toxins or uremic toxins themselves, such as trimethylamine N-Oxide (TMAO), p-cresyl sulfate (pCS), and indoxyl sulfate (IS), result from nutrient processing by intestinal microbes and can lead to renal and cardiovascular damage 12. Moreover, also originated from microbial nutrient conversion and implicated in various pathogenic processes in patients or animals with CKD are ammonium hydroxide, short-chain fatty acids (SCFAs), and secondary bile acid 13. Together, comprehensive metagenomic and metabolomic analyses, thus, maybe useful for a better understanding of CKD development through associated alterations in the gut ecosystem.

Even though 16S rRNA-based sequencing has made significant strides in medical microbiology, deep shotgun metagenomic sequencing is believed to offer more in-depth taxonomic characterization 14 and functional insights into the human microbiomes 15 than 16S rRNA gene sequencing. To date, studies of gut microbiome on CKD have been focused on either the advanced stages of disease 6-8, 16-18 or on early renal function decline 9. Here, we utilized an integrative approach to connect information on the gut microbiome with circulating host-microbe co-metabolite data by conducting shotgun metagenome sequencing of fecal samples and targeted profiling of serum bile acids, SCFAs (<6 carbon atoms), medium-chain fatty acids (MCFAs, 6-12 carbon atoms), and uremic toxins (pCS and IS) in a cohort of CKD patients with different severities and non-CKD controls. Our dual-omics analyses reveal several dysbiotic genes/pathways and species-level clades in robust associations with circulating metabolites and disease severities, which may be of etiological and diagnostic implications.

## Methods

### Study cohort and sample collection

In this cross-sectional study, 72 patients with CKD were enrolled in the Department of Nephrology, Chang Gung Memorial Hospital, Keelung, Taiwan, with the approval by the institutional review board (IRB: 201802061B0, 201802245B0, and 201900167B). CKD was defined as either the presence of proteinuria or an estimated glomerular filtration rate (eGFR, determined by using simplified Modification of Diet in Renal Disease equation) of less than 60 ml/min/1.73 m2 in two distinct appointments and classified into stage 1 to 5, according to the NKF/DOQI classification 19. To determine CKD stage more performatively, GFR was estimated by using two additional formulas, the Cockcroft-Gault (CG) 20 and CKD-EPI (Chronic Kidney Disease Epidemiology Collaboration) equation 21. For exploring the disease progression, patients were grouped into mild (stage 1 and 2, n=26), moderate (stage 3, n=26) and advanced (stage 4 and 5, n=20) CKD. In addition, 20 subjects with normal renal function and matched age, gender, and status of diabetes and hypertension were recruited for comparisons. All participants provided informed written consent at enrollment. Patients with malignancy, liver cirrhosis, intestinal operation, irritable bowel syndrome, cardiovascular disease (defined as myocardial infarction, documented Q wave on EKG, unstable angina, coronary artery disease with stenosis >75%, congestive heart failure with an ejection fraction <50% and cerebrovascular disease), active infection, concomitant use of probiotics, prebiotics or antibiotics, pregnancy or renal transplant recipients were excluded from the study. 5 mL of whole blood sample from each subject was withdrawn after 8 h of fasting, allowed to clot at room temperature for 30 min, and centrifuged at 1,500 ×g for 10 min. Aliquots of the resulting supernatant were stored at -80 °C until usage. 10 g of stool sample was harvested, placed in nucleic acid preservative (DNA/RNA Shield, Zymo Research), delivered to our research center within 24 to 48 h after collection, and stored at -80 °C until fecal DNA extraction. Within 7 days before sample collection, subjects were not allowed to take any supplement or food containing probiotics such as yogurt. All participants received diet interview and had regular 3-meals dietary pattern. 24-h urinary urea was measured to estimate dietary protein intake of each subject. Vegetarian or people on vegan diets were also excluded to avoid distortion on the dietary pattern of entire cohort.

### Shotgun metagenome sequencing

Fecal DNA was isolated by using the FastDNA SPIN Kit for Feces (MP Biomedical) and fragmented by using a Covaris M220 sonicator (Covaris, Inc.). The library preparation was conducted by using a TruSeq DNA sample preparation kit (Illumina) according to manufacturer's instructions, and at least 6 Gb of 150 bp pair-end (PE) reads per sample were generated on an Illumina HiSeq instrument.

### Metagenomic data processing and analysis

PE reads were merged and trimmed using in-house scripts to remove the Illumina adapters before short (<60 bp) or low-quality reads (containing more than 50 % of bases with a Q score =< 5) were excluded. Host sequences were then discarded by mapping the sequences against the reference genome (hg19) using BowTie2 22. The remaining set of clean reads was pooled and subject to metagenome assembly using MEGAHIT 23 to create contigs. Clean reads were mapped to contigs using BowTie2, and the mapping output was used for contig binning through MetaBAT 24. Qualified bins (>=50% completeness, <=5% contamination) determined by CheckM 25 were used for further analysis. Prediction of genes was performed from assembled contigs using MetaGeneMark 26. After dereplication, all non-redundant genes were used to construct the gene catalogue. Gene abundance in each sample was determined by alignment of the reads using BWA MEM against the gene catalogue and measuring the number of reads mapped to each gene sequence. The taxonomic assignment of individual open reading frames (ORFs) was used to annotate bins. A bin was assigned to the taxon to which more than 50% of its ORFs belong to. For constructing the species-level taxonomic profile, the relative abundance of a species was computed per sample, based on the number of reads assigned to the species divided by the total number of aligned reads in the sample. If multiple bins were annotated to the same species, their relative abundance was added up. Functional annotation was performed by mapping annotated ORFs to the Kyoto Encyclopedia of Genes and Genomes (KEGG) protein database using GhostKOALA 27 for labeling non-redundant genes with KEGG orthologue (KO) number. The relative abundance of genes from the same KO was summed up and used as the content of this KO in a sample to generate the KO profile.

### Targeted metabolomics profiling of serum samples

For targeted profiling of circulating SCFA and MCFA, 11 analyte standards (propionic acid, butyric acid, valeric acid, capric acid, caproic acid, pelargonic acid, isobutyric acid, isovaleric acid, heptanoic acid, acetic acid, and caprylic acid) were purchased from Sigma. 250 μL of internal standard solution containing 10% H_2_SO_4_ (Sigma) and 20 mg/L 2-methylvaleric acid (Dr. Ehrenstorfer GmbH) was added to 150 μL of serum samples. Samples were vortexed for 30 s, followed by oscillation for 10 min and centrifugation at 10,000 rpm, at 4 °C for 15 min, and left at -20 °C for 30 min before transferring the organic layer into glass vials for GC-MS analysis. GC-MS analysis was performed using an Agilent 7890B gas chromatograph system coupled with an Agilent 5977B mass spectrometer. 1 μL of the analyte was injected in split mode (5:1). Helium was used as the carrier gas in an HP-FFAP capillary column (30 m×250 μm×0.25 μm). The front inlet purge flow was 3 mL/min, and the gas flow rate through the column was 1 mL/min. The initial oven temperature was maintained at 80 °C for 1 min, then ramped to 150 °C at a rate of 5 °C/min and immediately raised to a final temperature of 230 °C at a rate of 40 °C/min and held for 12 min. The injection, transfer line, quad and ion source temperatures were 240 °C, 240 °C, 230 °C and 150 °C. Electron impact mode at 70 eV was used for electron ionization. The mass spectrometry data were acquired in Scan/SIM mode with a m/z range of 33-200 after a solvent delay of 5 min.

For targeted profiling of 41 circulating bile acids (**Table S1**), all analyte and internal standards (glycocholic acid-D4, deoxycholic acid-D6, and taurochenodeoxycholic acid-D4) were LC-MS grade and obtained from CNW Technologies. The stock solution of each individual standard was prepared in methanol at a concentration of 10 mmol/L, and the working solutions for each standard were obtained by a series of dilutions in methanol-acetonitrile-water (2:2:1, containing 0.1% formic acid). 100 *μ*L of serum sample was mixed with 400 *μ*L of extract solvent (acetonitrile-methanol, 1:1, containing 0.1% formic acid), vortexed for 30 s, sonicated for 10 min in ice-water bath, followed by incubation at -20°C for 1 h and centrifugation at 11000 rpm, at 4°C for 15 min. The supernatant was transferred to an autosampler vial for subsequent UHPLC-MS/MS analysis. UHPLC separation was performed in an Agilent 1290 Infinity series UHPLC System, equipped with a Waters ACQUITY UPLC BEH C18 column (150 × 2.1 mm, 1.7 *μ*m, Waters) (Agilent Technologies). The sample injection volume was 3 *μ*L. Eluents consist 0.1% acetic acid in water (phase A) and 0.1% acetic acid in acetonitrile (phase B). A 32-min elution gradient was performed as shown in **Table S2**. The MS analysis was conducted by using a Q Exactive Focus mass spectrometer (Thermo Fisher Scientific). Quantification was performed the parallel reaction monitoring (PRM) assay. The optimal MS parameters were as follows: spray voltage, +3500/-3100 V; sheath gas (N2) flow rate, 40 L/h; aux gas (N2) flow rate, 15 L/h; sweep gas (N2) flow rate, 0 L/h; aux gas (N2) temperature, 350 °C; capillary temperature, 320 °C. Our UHPLC-PRM-MS/MS analysis was conducted according to the US FDA guideline for bioanalytical method validation. For obtaining all calibration curves, the measurement was excluded from the calibration if the signal-to-noise ratio (S/N) was below 20, or accuracy of calibration was not within 80-120%. The S/N was used to determine the lower limits of detection (LLODs) and lower limits of quantitation (LLOQs). The LLODs and LLOQs were defined as the lowest analyte concentrations that could provide a S/N of 3 and 10, respectively.

Serum levels of free and total (sum of the free and protein-bound fraction) pCS and IS were analyzed with LC/ESI-MS (Thermo Fisher Scientific TRANSCEND and TSQ VANTAGE). Concentrations of free pCS and IS were measured in serum ultrafiltrates by using AmicoUltra 30 K filter (Millipore). 100 μL of samples were deproteinized by adding 4 parts of acetonitrile. Chromatographic separation was conducted at 40 °C using a Hypersil Gold aQ Column 1.9 μm (2.1x100mm). The separation was run for a total of 6 minutes using a gradient elution composed of solvent A (0.1% Formic acid) and solvent B (1mM NH4OAc +0.1% formic acid in 100% acetonitrile) as follows: from 95% A/5% B to 10% A/90% B. MS data were processed using the Xcaliber software package (Thermo Fisher Scientific).

### Measurement of lipopolysaccharide (LPS)

Serum levels of LPS were determined using a double antibody sandwich method (MBS266722, MyBioSource, San Diego, CA, USA) according to the manufacturer's protocol. Briefly, serum samples were diluted 1:250 in standard diluent and then incubated in the plate with immobilized antibodies for 90 min at 37 °C, washed twice by PBS, and then incubated with biotinylated antibodies for 60 min at 37 °C. After wishing, avidin-peroxidase conjugates were added to the well, and TMB substrate was used for coloring after reactant thoroughly washed out by PBS. The color depth was then measured with a microplate reader within 10 min.

### Statistical analysis

Differences in clinical indices among groups were determined using Student's t-test or Kruskal-Wallis test. α diversity (within-sample) estimated by the Simpson index based on the taxonomic profile of each sample and β diversity (between-sample) assessed by Bray-Curtis distance were compared by using Wilcoxon rank sum and Student's t test, respectively. Differentially elevated or depleted gut microbes and serum metabolites were evaluated by Wilcoxon rank sum test. The connection of microbes to host metabolites was assessed by Spearman's rank correlation, and the importance was corrected by using the Benjamini-Hochberg procedure. Statistically significant species for each group were evaluated by the linear discriminant analysis (LDA) of effect size (LEfSe) analysis, which employed the non-parametric factorial Kruskal-Wallis test, Wilcoxon rank sum test and LDA to identify differentially abundant biomarkers between two metadata classes. Differentially enriched KEGG modules of five selected pathways (carbohydrate metabolism, lipid metabolism, amino acid metabolism, glycan biosynthesis and metabolism, and biosynthesis of other secondary metabolites) in metabolism were identified according to their reporter score 28, calculated from the Z-scores of individual KO groups. A module with reporter score Z >1.5 (> 90% confidence according to a normal distribution) was considered as a significant dysbiosis module. To determine a panel of gut microbes and circulating metabolites (bile acids and SCFAs) for detecting CKD with different severities, two steps were conducted. In the 1^st^ step, Random Forests 29 models were used to rank each type of profiles independently and validated by 10-fold stratified cross-validation testing (500 trees, balanced class weight, max features=square root of all features). The optimal number of discriminatory markers for each type of profiles was determined using the recursive feature elimination method 30 with default parameters using five different random seeds. For each test, the performance of the model was examined by receiver operating characteristic (ROC) curves constructed by pROC 31 using five-fold cross-validation. In the 2^nd^ step, discriminatory makers of two profiles identified in the previous step were pooled. Ranking, determination of the optimal number of features, and testing for the performance of the combination of gut bacterial and serum metabolic markers were conducted in the same method. Unless otherwise stated, all statistical tests are two-tailed, and a p < 0.05 is considered statistically significant.

## Results

### Study design and subject characteristics

To explore the association of gut microbes with host circulating metabolites in CKD, we collected fecal DNA and serum samples from a cross-sectional cohort of 92 subjects, comprising 72 cases (26 mild, 26 moderate and 20 advanced CKD) and 20 non-CKD controls for shotgun metagenome sequencing and targeted metabolomics analyses, respectively (**Figure S1**). Since specific gut dysbiosis has been associated with diabetes 15 and hypertension 32, two most common comorbidities in CKD patients, non-CKD controls with normal renal function and matched age, gender, and status of diabetes mellitus and hypertension (**Table 1**) were recruited to exclude potential confounding factors. The levels of blood urea nitrogen, serum creatinine, and eGFR mirror the disease severity among groups.

### Metagenomic and metabolomic signatures of CKD with different disease severities

**Figure 1:**
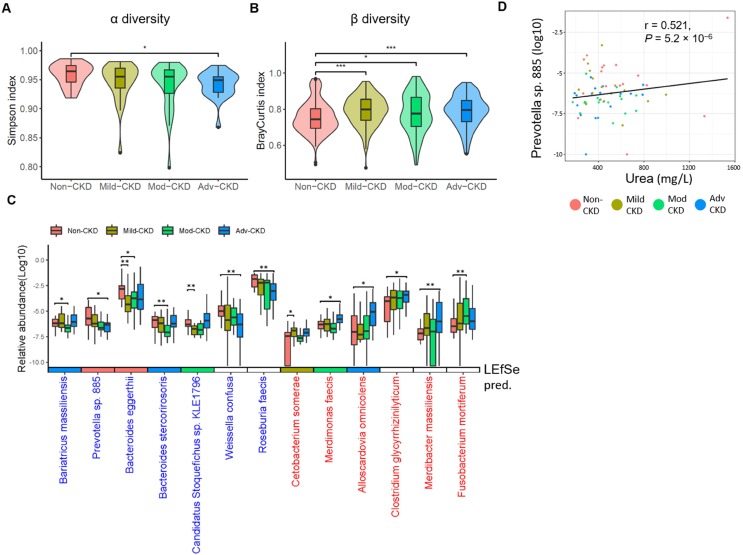
** Severity-specific changes in bacterial diversities and taxonomic signatures.** α-diversity **(A)** and β-diversity **(B)** among the groups were estimated by the Simpson and Bray-Curtis similarity indexes, respectively. The box-plot shows the median, the 25th, and the 75th percentile in each group. Simpson index was analyzed using Wilcoxon rank sum test, and Bray-Curtis distance between groups was calculated by Student's t test. *,* p* <0.05; ***,* p* <0.001; Mod-CKD (moderate CKD); Adv-CKD (advanced CKD). **(C)** Relative abundances of species among different groups were analyzed by Wilcoxon rank sum test. **, *p* < 0.005; *, *p* < 0.01. Species in red and blue denote CKD-enriched and -decreased markers, and the color in the box below represents significant species for the indicated group predicted by using LEfSe. **(D)** The relationship between *Prevotella sp. 885* and urinary urea excretion determined by Pearson correlation (r = 0.521, *P* = 5.2 × 10^-6^).

To comprehensively explore gut microbiome linked to CKD progression, shotgun sequence data of fecal samples were processed using our in-house pipeline, performing quality control checks, filtering, and metagenome assembly (**Figure S2**). We first compared intestinal microbial diversity across groups. A descending trend in within-sample species richness (α diversity) was detected with the disease severity, as the species richness in advanced CKD was significantly lower than that in non-CKD controls (**Figure 1A**). On the contrary, we observed higher sample-to-sample dissimilarities (β diversity) in CKD microbiomes (**Figure 1B**), indicating a more heterogeneous community structure among different CKD groups than in controls. To dissect the detailed taxonomic features, we performed a severity-specific comparison where each of CKD severity groups was compared to the non-CKD controls, together with LEfSe based on the abundance of 213 species-level clades. In total, seven and six species showed significant decrease and elevation (p<0.01), respectively, in at least one of CKD severity groups (**Figure 1C**), most of which were consistently selected by LEfSe (**Figure S3**). We noticed two patterns of significant species-level changes: one altered (elevated or decreased) across early to advanced stages, whereas the other altered only in particular stage(s). The former was characterized by four decreased (*Prevotella sp. 885*, *Weissella confuse*, *Roseburia faecis*, and *Bacteroides eggerthii*) and three elevated species (*Alloscardovia omnicolens*, *Merdibacter massiliensis*, and* Clostridium glycyrrhizinilyticum*). The latter was characterized by *Cetobacterium somerae* (mild CKD), *Candidatus Stoquefichus sp. KLE1796* (mild CKD), *Fusobacterium mortiferum* (moderate CKD), *Bariatricus massiliensis* (moderate CKD), *Bacteroides stercorirosoris* (moderate CKD), and *Merdimonas faecis* (advanced CKD). Further analysis revealed that the decline in the abundance of *Prevotella sp. 885* was correlated with urea excretion in the urine of our cohorts (**Figure 1D**), implicating a role of the gut-kidney axis in renal impairment.

**Figure 2:**
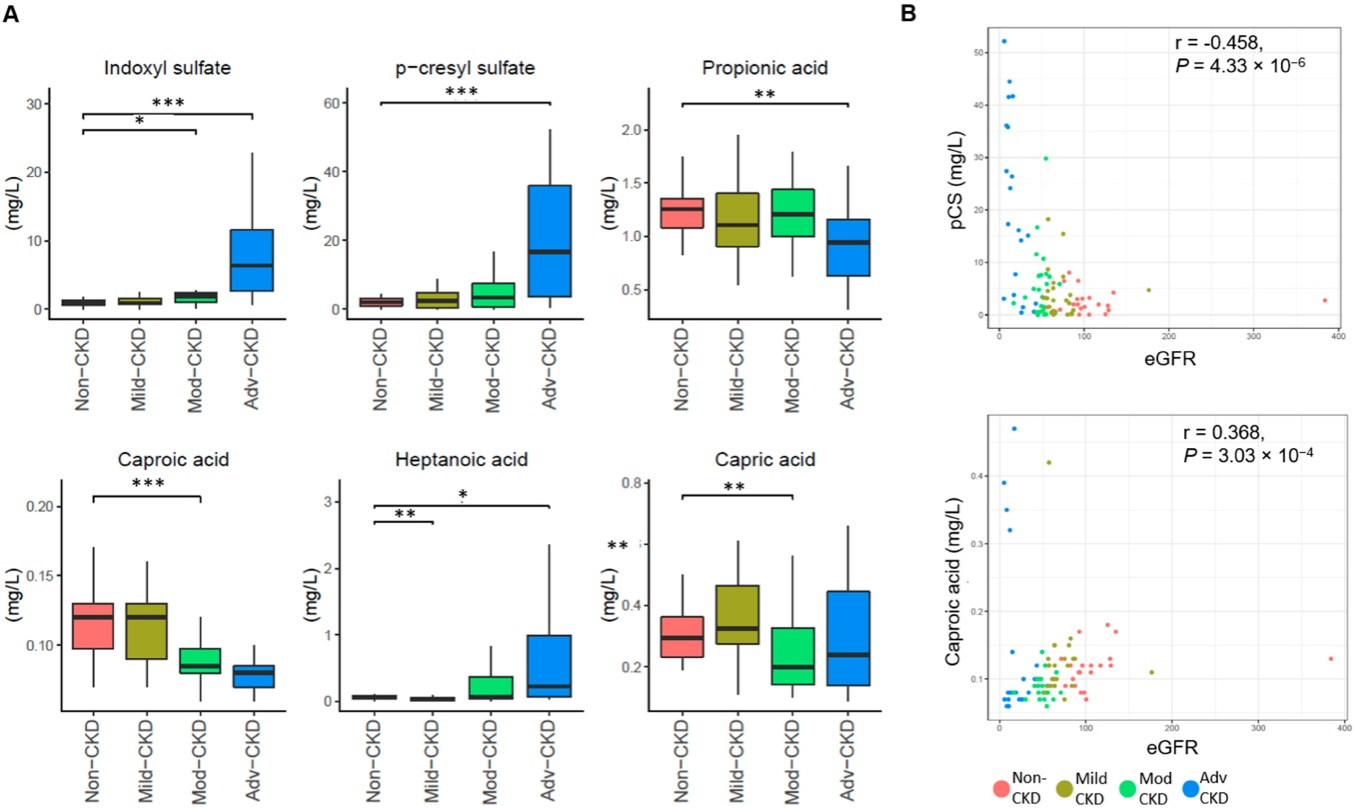
** Severity-specific changes in circulating metabolic signatures of CKD. (A)** Levels of metabolites among different groups were analyzed by Wilcoxon rank sum test. ***, *p* < 0.001; **, *p* < 0.005; *, *p* < 0.01. **(B)** The correlation of caproic acid (Spearman's correlation, r = 0.368, *P* = 3.03 × 10^-4^) and pCS (r = -0.458, *P* = 4.33 × 10^-6^) with the estimated glomerular filtration rate (eGFR).

To examine CKD severity-specific changes in the host-microbe co-metabolites, we performed targeted metabolomic profiling of 11 saturated fatty acids, 41 bile acids, and two uremic solutes (IS and pCS). Compared to the non-CKD controls, four fatty acids (capric acid, caproic acid propionic acid, and heptanoic acid) were significantly decreased (p<0.01) in at least one of CKD severity groups and two nephrotoxins (IS and pCS) were significantly elevated at the advanced stage (**Figure 2A**). Levels of caproic acid and pCS were positively and inversely correlated associated with the glomerular filtration rate, respectively (**Figure 2B**). In addition, marginally significant depletion of two bile acids (glycodeoxycholic acid and glycochenodeoxycholic acid) and three additional fatty acids (valeric acid, acetic acid, and caprylic acid) was noted at the late stage of CKD (**Figure S4**).

### Associations of gut microbial species with circulating host-microbe co-metabolites

**Figure 3:**
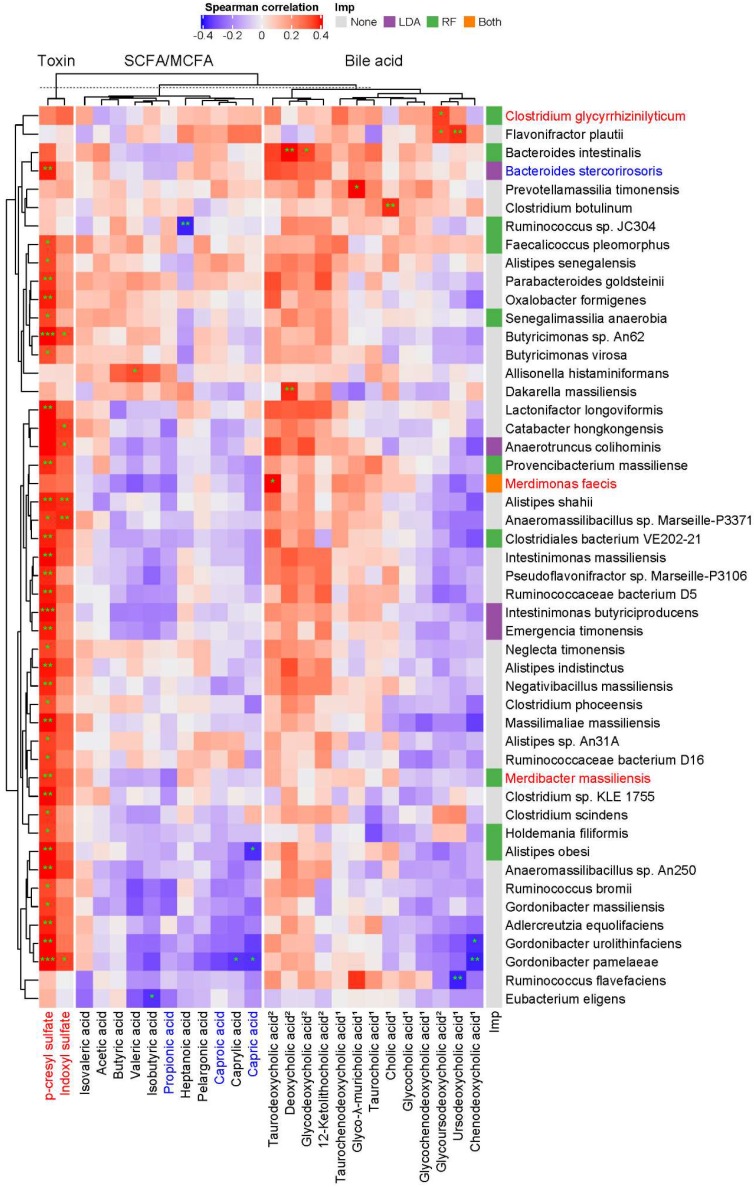
** Potentially mechanistic associations of gut microbes with circulating metabolites.** Spearman's correlation of serum metabolites with gut microbes, specifically those selected by Random Forests model (RF) against the glomerular filtration rate, LEfSe (LDA, *p*<0.05), or both (* q<0.1, ** q<0.05, *** q<0.01). Species and metabolites in red and blue denote the CKD-enriched and -decreased markers, respectively. Numbers in superscript, 1 and 2, indicate primary and secondary bile acids, respectively. Imp., Model of Importance Variable. Data are shown as metabolites were detectable in at least 50% of samples.

To examine whether specific intestinal microbes are responsible for a mechanistic relationship relating the abundance of microorganism and metabolite that is perturbed during the progression of CKD, a large-scale association discovery of 213 species with circulating metabolites was assessed. We identified a set of species that is positively correlated with the levels of pCS (**Figure 3**), many of which consistently were selected as biomarkers by LEfSe or Random Forests regression model against the eGFR (**Figure S3**), suggesting a potential involvement of such associations in CKD pathogenesis.

Although not statistically significant, the abundance of these pCS-associated gut microbes, in general, was positively correlated with the levels of many secondary bile acids detected whereas inversely associated with that of many primary bile acids, SCFAs and MCFAs, implicating a connection of these fatty acids and bile acids with renal function. Notably, *Alistipes obesi* and *Gordonibacter pamelaeae* were positively associated with the levels of pCS but anti-correlated with that of a significantly decreased MCFA, capric acid. These results reveal a host-microbe-metabolite network in CKD progression.

### Functional characterization of gut microbiome across different CKD subgroups

**Figure 4:**
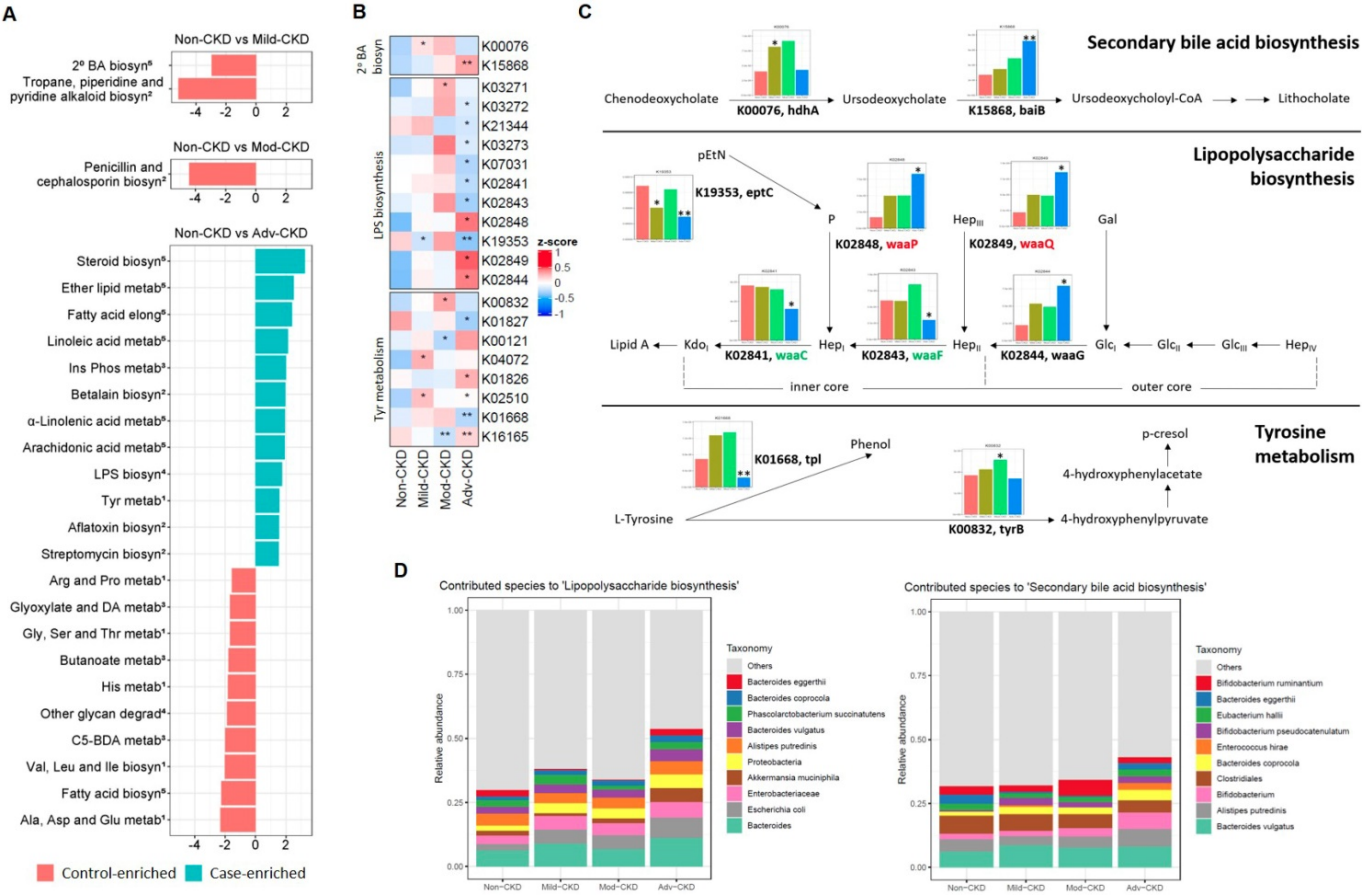
** CKD-associated changes in gut microbial function and their metabolomic associations. (A)** Comparisons of the relative abundance of KEGG modules (from 5 selected pathways in metabolism: ^1^amino acid metabolism; ^2^biosynthesis of other secondary metabolites; ^3^carbohydrate metabolism; ^4^glycan biosynthesis and metabolism; ^5^lipid metabolism) between each CKD severity group and controls by using the reporter score. 2^o^ BA biosyn, secondary bile acid biosynthesis; Ins Phos metab, Inositol phosphate metabolism; Glyoxylate and DA metab, Glyoxylate and dicarboxylate metabolism; C5-BDA metab, C5-branched dibasic acid metabolism. **(B)** Abundance of KO genes involved in representative pathway modules showed significant difference(s) in at least one CKD severity group. KO genes with the prevalence of 5% or higher are shown. Significant changes are denoted as follows: **, *P* < 0.01; *, *P* < 0.05, by Wilcoxon rank sum test. **(C)** Changes in gene abundance in **(B)** are shown in a simplified pathway presentation. For LPS biosynthesis, the structure and biosynthesis of the core oligosaccharide of LPS in *E. coli* K-12 are shown. Glycosyltransferases that construct the inner core backbone are shown in green; enzymes that modify the structure of inner core are shown in red. The inner core, containing residues of 3-deoxy-D-manno-octulosonic acid (Kdo) and L-glycero-D-manno-heptose (Hep), is often decorated with phosphate (P), while the outer core region typically contains glucose (Glc) and Hep. Gal, galactose; pEtN, phosphorylethanolamine. **(D)** Relative abundance of bacterial species associated with representative KEGG modules. Top 10 dominant bacterial species are denoted by color (right), and the remaining ones are assigned as others.

We next investigated the functional capacities of gut microbiome across different CKD severity groups. By mapping our bacterial gene catalog onto the KEGG database, we obtained 7,513 KEGG orthologs (KOs) present in at least five samples for subsequent analyses. With a focus on five selected pathways relevant to diet metabolism, a total of 25 differentially enriched KEGG modules were identified as compared to the non-CKD controls (**Figure 4A**), most of which were detected at the advanced stage (12 case-enriched and 10 control-enriched). Among these dysbiotic modules at the advanced stage, genes involved in lipid metabolism, such as steroid biosynthesis, ether lipid metabolism, and polyunsaturated fatty acid metabolism, were enriched in the CKD microbiome, and while those associated with carbohydrate and amino acid metabolism were more abundant in the controls. Of note, genes implicated in lipopolysaccharide (LPS) biosynthesis were enriched in the cases of advanced stage, suggesting that an elevation in inflammatory responses may contribute to renal health in CKD. Moreover, genes related to secondary bile acid biosynthesis were differentially enriched at the early stage, indicating that conversion of primary bile acids to secondary bile acids by intestinal microbes takes place in early renal function decline.

Of particular interest in induction of host inflammation and nephrotoxic metabolites, we examined microbial gene abundances to check the roles of the microorganisms in selected dysbiosis modules, including LPS biosynthesis, secondary bile acid biosynthesis, and tyrosine metabolism (**Figure 4B**). We noted that the abundance of genes responsible for biosynthesis of the core oligosaccharide of LPS was altered during CKD progression (**Figure 4C**). Specifically, genes encoding enzymes that modify the inner core backbone (WaaP, WaaQ) were enriched, whereas those encoding glycosyltransferases that construct the inner core backbone (WaaC, WaaF) were decreased at the advanced stage of CKD, suggesting alterations in the stability and structure of LPS in CKD pathophysiology. In addition, we observed that the abundance of genes related to conversion of primary (chenodeoxycholate) to secondary bile acid was elevated in CKD microbiome. Notably, the abundance of an elevated gene (K00076, hdhA) was significantly correlated with serum levels of an intermediate metabolic product, ursodeoxycholate (**Figure S5**), highlighting a role of gut microbiome in the elevation of serum bile acids in patients with CKD. For tyrosine metabolism (a case-enriched module at the advanced stage), we found that alterations in gene abundance (enrichment of tyrB and depletion for tpl) at late stages favor the production of 4-hydroxyphenylpyruvate, a precursor of p-cresol. This in part accounts for the increase in serum pCS levels observed at the late stages of renal dysfunction (**Figure 2A**).

Further analyses of the predominant species that contribute to these dysbiosis modules (**Figure 4D**) revealed that augmented LPS biosynthesis was in part attributed by an increase in the abundance of *Escherichia coli* and other species of the family *Enterobacteriaceae*. One significantly-decreased species, *Bacteroides eggerthii*, to some extent contributed to secondary bile acid biosynthesis at the early stage. These findings point out an involvement of gut dysbiosis and its associated functional shift in regulating inflammatory reactions and metabolic activities.

### Dual-omics signatures differentiate CKD from non-CKD controls

**Figure 5:**
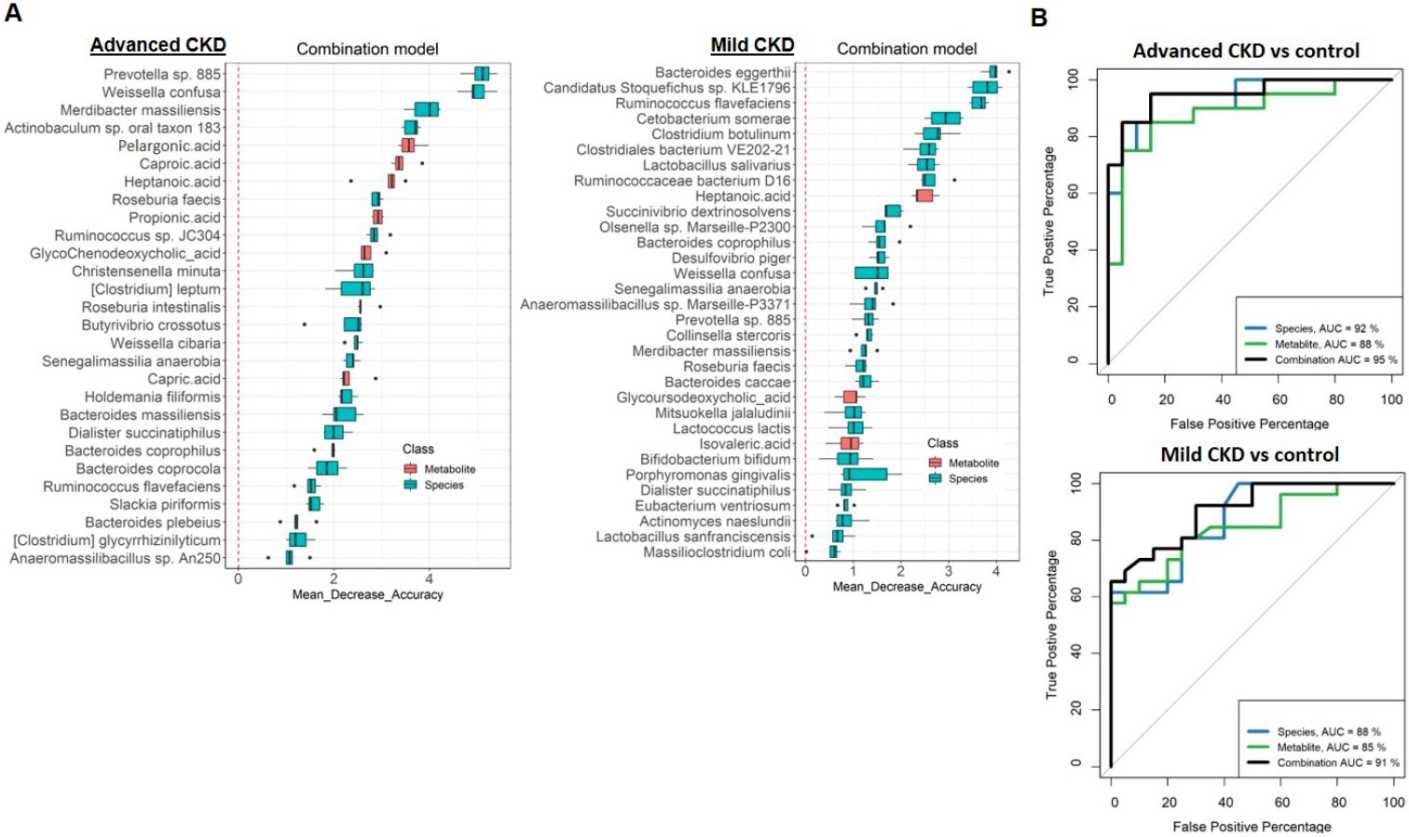
** (A)** Metagenomic and metabolomic markers for detecting patients with advanced CKD (right, n=20) and early-stage CKD (left, n=26) from the controls (n=28) identified from Random Forests classifiers based on the combination of dual-omics markers. Markers are ranked in descending order of their importance to the accuracy of the model. The boxes represent 25th-75th percentiles, and black lines indicate the median. **(B)** ROC curves depict trade-offs between true and false positive rates for detecting patients with advanced and early-stage CKD as classification stringency varies. AUC, the total area under the ROC curve.

In order to test the potential of gut metagenomic and serum metabolomic parameters to serve as diagnostic markers, we constructed Random Forests regression models based on species only, metabolite only (exclusion of two highly discriminatory nephrotoxins, IS and pCS) (**Figure S6 and S7**), or a combination of both (**Figure 5A and S8**) to discriminate cases with different severities from non-CKD controls. Comparison of the classification potential among the three models revealed that the combination model outperformed the species or metabolite only model in the discrimination of three CKD severity groups from the controls (**Figure 5B and S8**), indicating an improvement of the discriminative ability by using dual-omics signatures. Use of the combination model achieved a total area under the ROC curve (AUC) of 0.95 and 0.90 to detect a patient with advanced and mild CKD, respectively. Different from the classification of moderate CKD with high-ranking features being MCFAs (caproic acid, the top one; capric acid, the second) (**Figure S6**), highly discriminatory markers of mild and advanced CKD classification are bacterial species (*Bacteroides eggerthii* and *Prevotella sp. 885* are the top-ranking features for mild and advanced CKD classification, respectively). In addition, several individual markers with clinical validity for specific CKD severity groups were identified (**Table 2**) by evaluating those differentially-abundant species (**Figure 1**) and metabolites (**Figure 2**) individually. Among them, a greater AUC in discriminating early-stage CKD from the controls was achieved for *Bacteroides eggerthii* (AUC, 0.80; 95% CI, 0.67-0.93) than the use of urine protein/creatinine ratio (AUC, 0.64) or serum urea (AUC, 0.72). Our results reveal promising avenues for early diagnosis of CKD via specific intestinal microbes.

## Discussion

Dysbiosis of intestinal microbiome and its associated changes in host metabolism and inflammation may dictate the development of CKD. Nevertheless, recent studies of human gut microbiome on CKD have been 16S rRNA-based and mostly focused on the advanced stage of disease 6-8, 16-18. Therefore, in-depth taxonomic and functional characterization of CKD microbiome is highly warranted. Here, we identified specific gut dysbiosis at the species level and its associated functional shift in strong associations with circulating metabolites at different stages of CKD. These connections highlight a potential host-microbe-metabolite interaction in the progression of renal dysfunction.

In the compositional alterations of gut microbiota, two patterns of significant species-level changes were observed. One is featured by enrichment or decrease across early to advanced stages, whereas the other altered only in particular stage(s). Among these differentially abundant microbes, *Bacteroides eggerthii* and *Prevotella sp. 885* are the top discriminatory features for mild and advanced CKD classification, respectively (**Figure 5A**). Specifically, in addition to *Prevotella sp. 885*, two decreased species (*Weissella confuse* and *Roseburia faecis*) and two elevated species (*Merdibacter massiliensis*, and* Clostridium glycyrrhizinilyticum*) across early to advanced stages were found to be highly correlated with disease progression and capable of distinguishing non-CKD controls from patients with advanced CKD. Notably, other than *Bacteroides eggerthii*, another two species, *Cetobacterium somerae* (elevated in mild CKD) and *Candidatus Stoquefichus sp. KLE1796* (decreased in mild CKD), were identified as top-discriminatory species between non-CKD controls and CKD patients at the early stage. Due to the initial asymptomatic characteristics of CKD, renal dysfunction might become irreversible after years, eventually progressing into ESRD if not early diagnosed. We demonstrated that in addition to the use of a selected panel of taxonomical and metabolic markers, *Bacteroides eggerthii* individually yielded superb performance of differentiating the controls from early-stage CKD. Moreover, *Prevotella sp. 885* mirrored the disease progression of CKD and was correlated with urea excretion, indicative of protein uptake and renal function. These results implicate specific gut microorganisms as a useful biomarker for early diagnosis and prognosis monitoring of CKD.

In searching circulating metabolic signatures of CKD, it is consistent with other studies 12, 33 that an elevation of uremic solutes (IS and pCS) and a depletion of SCFAs (propionic acid, valeric acid, and acetic acid) were observed. Among these SCFAs, propionic acid was significantly decreased at late stages and detected to be highly discriminatory between non-CKD controls and patients with advanced CKD (**Figure 5A**). Notably, we identified a number of MCFAs (capric acid, caproic acid, and caprylic acid) that was enriched in non-CKD controls. MCFAs have been shown to exhibit antimicrobial properties against bacteria and viruses 34 and to be significantly decreased in the fecal samples of patients with inflammatory bowel diseases (IBDs) 35. Here, levels of caproic acid, predicted as the top-discriminatory metabolites (besides IS and pCS) in moderate and advanced CKD, were highly correlated with eGFR, indicating its beneficial effect on intestinal inflammation and potential diagnostic value on renal dysfunction. We also observed fluctuations in serum levels of bile acids although the changes did not meet our significance threshold. Bile acids, which regulate numerous metabolic pathways in the host via the nuclear farnesoid X receptor and the G protein-coupled receptor 5 36, were differentially abundant in serum of ESRD patients 37. Our results together with others' provide potential avenues for the improvement of CKD diagnosis and modality through these host-microbe co-metabolites.

Moreover, we identified gut microbial species linked to alterations in serum metabolites of the host. Intriguingly, based on the associations between each microbe and metabolite, MCFAs and SCFAs can be grouped into two distinct clusters, and secondary bile acids can be separated from primary bile acids (**Figure 3**). Likely consistent with a mechanistic relationship relating the abundance of the species and metabolite that is perturbed during the development of CKD, these microbe-metabolite associations could indicate that the species produces that metabolite, or the metabolite promotes/inhibits the growth of that species. For instance, a positive correlation between *Clostridium glycyrrhizinilyticum* (a significantly elevated species belonging to cluster XIVa of the genus *Clostridium*) and a secondary bile acid, glycoursodeoxycholic acid (GUDCA), was detected. In humans, *Clostridium* cluster XIVa was known to produce secondary bile acids 36, and oral administration of GUDCA enhanced the growth of *Clostridium* cluster XIVa in mouse with colitis 38. In addition, *Bacteroides intestinalis*, a highly discriminatory species predicted by Random Forests analysis, was positively associated with serum levels of deoxycholic acid (DCA). Biochemical testing has shown that *Bacteroides intestinalis* converts cholic acid into its 7-oxo derivative, 7-oxo-DCA 39. Another relevant example is the detection of association between *Alistipes shahii* (an indole-positive species 40) and IS. These mutual connections reflect the mechanistic linkage within the gut ecosystem, which collectively may serve as a determinant in CKD pathogenesis.

The functional capacities of gut microbiome in CKD are largely unexplored. Here, with a focus on pathways relevant to microbial conversion of diet, we found that pathway modules associated with lipid metabolism, such as steroid biosynthesis, ether lipid metabolism, and polyunsaturated fatty acid metabolism, were enriched in the CKD microbiome at the advanced stage. Metabolic syndrome, which is commonly associated with dyslipidemia, remains a key etiological parameter of CKD. Among diverse lipid types, ether lipids, enriched in lipid raft microdomains of cell membranes, play a vital role in membrane dynamics and cellular signaling 41. Decreased serum levels of ether lipids have been implicated in hypertension 42, obesity 43, and diabetes 44. Regardless of the underlying mechanism, our finding suggests that, for the first time, breakdown of ether lipids by intestinal microbiota may serve as a marker for diagnosis of CKD or other comorbidities. In addition to modules in lipid metabolism, microbial genes related to LPS biosynthesis were more abundant in cases of the advanced stage. This finding is consistent with the observation that serum levels of LPS increased with the degree of renal impairment and peaked at the advanced stage of CKD (**Figure S9**). A crucial characteristic of LPS structure is the presence of phosphoryl substituents on the heptose residues of the inner core backbone (**Figure 4C**). Such modifications of LPS are essential to bacterial outer membrane stability 45 and its completed virulence 46, likely because the negative charge allows neighboring LPS molecules to be cross-linked by divalent cations. Of note, genes encoding enzymes that modify the inner core backbone (WaaP, WaaQ) were enriched in the advanced-stage CKD microbiome, indicating a sustained elevation of inflammatory responses via enhanced bacterial outer membrane integrity. Through analyzing the predominant species that contribute to the dysbiotic module in CKD microbiome, we found that augmented LPS biosynthesis was in part attributed by an increase in *Escherichia coli* and other species of the family *Enterobacteriaceae* and of the phylum *Proteobacteria*. These species contain specific enzymes to produce LPS with hexa-acylated lipid A moiety 47, as hexa-acylated LPS (6 acyl) is 100-fold more immunostimulatory when bound to TLR4 than LPS that is penta-acylated (5 acyl) 48. These results implicate an induction of endotoxins, in particular, a more immunostimulatory version, in CKD pathophysiology. Furthermore, another intriguing finding is the enrichment for the module of aflatoxin biosynthesis in CKD microbiome. Nephrotoxic effects of aflatoxin have been observed in humans 49 and experimental rodent models 50, 51. Consistently, our metagenomics analyses support a role of this gut flora-derived secondary metabolite in CKD etiology. Taken together, our findings in this study recapitulate a functional involvement of gut dysbiosis in orchestrating inflammatory and metabolic reactions in the development of CKD.

To define the functionality of gut microbiome and its association with the host in CKD, additional work is needed to address several limitations of the present study. One drawback is that a replication cohort is lacking although many lines of our metagenomic and metabolomic results are consistent with previous findings from others. Also unavailable is the information regarding fiber intake as consumption of dietary fibers may modify gut microbial ecology 52. Another issue is that the details of many associations identified remain to be interpreted and determined due to limited knowledge from relevant studies to date. Ideally, validation of these microbe-metabolite interactions and their role in CKD pathogenesis can be addressed through further *in vivo* experiments. Such attempts are often laborious; thus, computational exploration of putative links detected in the study by integration of dual-omics profiling could be an important aid.

In conclusion, our findings establish a comprehensive understanding in the relationship between the intestinal microbiota at the species level and host metabolism across different stages of CKD, providing potential avenues for microbiome-based diagnosis and modality of renal impairment.

## Supplementary Material

Supplementary figures and tables.

## Figures and Tables

**Table 1:** Baseline characteristics of study population

	Non-CKD (n=20)	Mild CKD (n=26)	Moderate CKD (n=26)	Advanced CKD (n=20)
Age, years	64.00 ± 7.06	62.69 ± 3.89	64.04 ± 6.21	65.00 ± 5.94
Male gender, n (%)	8 (40%)	11 (42.3%)	17 (65.4%)	8 (40%)
Diabetes, n (%)	9 (45%)	14 (53.8%)	11 (42.3%)	9 (45%)
Hypertension, n (%)	14 (70%)	22 (84.6%)	20 (76.9%)	19 (95%)
Diastolic pressure, mm Hg	73.95 ± 9.71	73.77 ± 11.13	75.58 ± 8.65	76.70 ± 12.53
Systolic pressure, mm Hg	128.75 ± 18.98	130.46 ± 16.24	128.96m± 13.88	137.35 ± 11.71
Body mass index, Kg/m^2^	24.35 ± 2.84	27.33 ± 3.52	26.85 ± 4.16	25.33 ± 3.35
Waist, cm	83.62 ± 8.61	92.58 ± 11.95	89.15 ± 7.41	86.12 ± 9.55
Blood urea nitrogen, mg/dL	13.35 ± 3.50	16.35 ± 5.04	20.15 ± 6.143*	55.10 ± 21.83*
Serum creatinine, mg/dL	0.69 ± 0.15	0.95 ± 0.23*	1.40 ± 0.52*	3.814 ± 1.89*
Estimated GFR, mL/min/m^2^, MDRD	114.93 ± 65.82	72.24 ± 23.89*	49.96 ± 10.65*	18.54 ± 11.20*
Estimated GFR, mL/min/m^2,^ CG	89.38 ± 21.64	74.58 ± 20.90*	53.14 ± 12.87*	20.55 ± 12.70*
Estimated GFR, mL/min/m^2^, CKD-EPI	95.10 ± 9.09	74.48 ± 15.29*	52.27 ± 11.44*	18.63 ± 12.10*
Urine protein-creatinine ratio, g/g#	89.63 (92.55)	126.82 (414.95)	103.29 (168.19)	1331.50 (3533.79)*
Hemoglobin, g/dL	13.35 ± 0.86	13.88 ± 1.30	12.99 ± 1.25	10.20 ± 1.94*
Serum albumin, mg/dL	4.49 ± 0.27	4.57 ± 0.25	4.53 ± 0.289	4.20 ± 0.62
Total cholesterol, mg/dL	188.35 ± 31.17	190.767 ± 24.78	171.82 ± 30.53	198.25 ± 50.75
LDL-cholesterol, mg/dL	107.68 ± 30.40	110.26 ± 22.65	96.30 ± 25.94	108.12 ± 36.94
Triglyceride, mg/dL	154.10 ± 98.12	133.69 ± 70.10	139.19 ± 80.17	185.35 ± 168.22
hs-CRP, mg/L#	0.90 (1.52)	1.31 (2.15)	1.18 (1.54)	2.57 (9.86)
Estimated protein intake, g/day	77.26 ± 24.11	76.56 ± 22.08	56.36 ± 20.745	60.67 ± 19.63
Phosphate binder, n (%)	0	0	0	0
Potassium chelator, n (%)	0	0	0	2 (10%)

Data are expressed in mean (SD) or median (interquartile range)#. Abbreviation: CKD, chronic kidney disease; GFR, glomerular filtration rate; CG, Cockcroft-Gault; CKD-EPI, Chronic Kidney Disease Epidemiology Collaboration; hs-CRP, high sensitive C reactive protein. * p < 0.005 vs non-CKD. Estimated protein intake (g/day) = 6.25 × [Urine urea nitrogen (g/d) + 30 mg/kg/d × Weight (kg)].

**Table 2:** Clinical validity for potential biomarkers in discriminating the controls from CKD patients with different severities.

Biomarker	Comparisons of different CKD severities							
	Non-CKD (20) vs. mild CKD (26)			Non-CKD (20) vs. advanced CKD (20)			Non-CKD (20) vs. overall CKD (72)	
	AUC (95% CI)	P value/Pc value		AUC (95% CI)	P value/Pc value		AUC (95% CI)	P value/Pc value
**Gut bacterial species**								
Bacteroides eggerthii↓	0.80 (0.67-0.93)	0.00026/**0.0034**		0.67 (0.49-0.85)	0.068/0.884		0.72 (0.61-0.84)	0.0013/**0.0169**
Alloscardovia omnicolens↑	0.51 (0.34-0.68)	0.92/11.96		0.77 (0.61-0.92)	0.0038/**0.0494**		0.58 (0.45-0.71)	0.24/3.12
**Serum metabolite**								
Indoxyl sulfate↑	0.63 (0.47-0.79)	0.12/0.72		0.94 (0.85-1)	1.7E-06/**1.02E-05**		0.74 (0.64-0.84)	0.00057/**0.00342**
p-cresyl sulfate↑	0.56 (0.40-0.73)	0.47/2.82		0.91 (0.81-1)	1.4E-06/**8.4E-06**		0.68 (0.56-0.79)	0.012/0.072
Caproic acid↓	0.55 (0.39-0.72)	0.52/3.12		0.67 (0.47-0.87)	0.066/0.396		0.68 (0.57-0.80)	0.0083/**0.0498**
**Conventional biomarker**								
Urine protein-creatinine ratio	0.64 (0.48-0.80)	0.106		0.96 (0.91-1)	<0.001		0.69 (0.59-0.80)	0.007
Serum urea	0.72 (0.58-0.87)	0.0078		1 (0.99-1)	<0.001		0.84 (0.76-0.92)	<0.001

CKD, chronic kidney disease; AUC, the total area under the ROC curve. Corrected P (Pc) values were adjusted by using Bonferroni's correction (n=13, for species; n=6, for metabolite).↑and↓indicate an ascending and descending trend of markers' abundance with the disease progression, respectively.

## Abbreviations

CKD: chronic kidney disease
ESRD: end-stage renal disease
TMAO: trimethylamine N-Oxide
pCS: p-cresyl sulfate
IS: indoxyl sulfate
SCFA: short-chain fatty acid
MCFA: medium-chain fatty acid
eGFR: estimated glomerular filtration rate
ORF: open reading frame
KEGG: Kyoto Encyclopedia of Genes and Genomes
KO: KEGG orthologue
LEfSe: linear discriminant analysis of effect size
ROC: receiver operating characteristic
LPS: lipopolysaccharide
AUC: area under the ROC curve
