# Supplementary material for: Integrative metagenomic and metabolomic analyses reveal severity-specific signatures of gut microbiota in chronic kidney disease
Source: Theranostics. 2020 Apr 6;10(12):5398–411. doi: 10.7150/thno.41725 (PMC7196299; doi:10.7150/thno.41725)
Supplement: Supplementary file 1 — Supplementary figures and tables. [file thnov10p5398s1.pdf]

Integrative metagenomic and metabolomic analyses reveal severity-specific signatures of gut microbiota in chronic kidney disease

I-Wen Wu[1, 2], MD; Sheng-Siang Gao[3], MS; Hsin-Cheng Chou[3], BS; Huang-Yu Yang[2,4,5], MD, PhD; Lun-Ching Chang[6], PhD; Yu-Lun Kuo[7], PhD; Michael Cong Vinh Dinh[8], BS; Wen-Hung Chung[9], MD, PhD; Chi-Wei Yang[2, 4], MD; Hsin-Chih Lai[10,11,12], PhD; Wen-Ping Hsieh[3], PhD; Shih-Chi Su[9,#], PhD

[1]Department of Nephrology, Chang Gung Memorial Hospital, Keelung, Taiwan

[2]College of Medicine, Chang Gung University, Taoyuan, Taiwan

[3]Institute of Statistics, National Tsing-Hua University, Hsinchu, Taiwan

[4]Kidney Research Center, Department of Nephrology, Chang Gung Memorial Hospital, Linkuo, Taiwan

[5]Department of Health Policy and Management, Johns Hopkins Bloomberg School of Public Health, Baltimore, Maryland, US

[6]Department of Mathematical Sciences, Florida Atlantic University, Florida, US

[7]Biotools, Co., Ltd, New Taipei City, Taiwan

[8]Department of Computer Science, Florida Atlantic University, Florida, US

[9]Whole-Genome Research Core Laboratory of Human Diseases, Chang Gung Memorial Hospital, Keelung, Taiwan

[10]Department of Medical Biotechnology and Laboratory Science, and Microbiota Research Center, College of Medicine, Chang Gung University, Taoyuan, Taiwan

[11]Central Research Laboratory, XiaMen Chang Gung Hospital, XiaMen, China

[12]Department of Laboratory Medicine, Chang Gung Memorial hospital, Linkuo, Taiwan

#Corresponding author: Shih-Chi Su, PhD

Whole-Genome Research Core Laboratory of Human Diseases, Chang Gung Memorial Hospital, Keelung, Taiwan.

222, Mai-Chin Road, Keelung 20401, Taiwan; Phone: 886-2-24329292-3388; Fax: +886-2-27191623; Email: ssu1@cgmh.org.tw

Supplemental information

Shotgun metagenome sequencing of stool DNA and taxonomic profiling

To obtain metagenomic profiles, we sequenced 92 DNA samples from stool to 7.09 gigabyte (Gb) per sample in average. Following quality control and removal of host sequence reads, an average of 6.51 Gb of microbial reads were collected per sample (ranging from 5.81 to 9.44 Gb). The bacterial sequencing reads from all 92 samples were pooled and *de novo* assembled into a set of 4,685,777 contigs that together comprise the metagenome (**Table S3**), with a N50 of 2,650 bp. The total length of assembled contigs is 4153.9 Mbp (**Figure S2A**), and the GC content of which is 47.45% (**Figure S2B**). Clustering of assembled contigs generated 904 phylogenetic bins. A total of 357 bins was assigned to the species level, from which 213 unique species-level clades were annotated (**Table S4**).

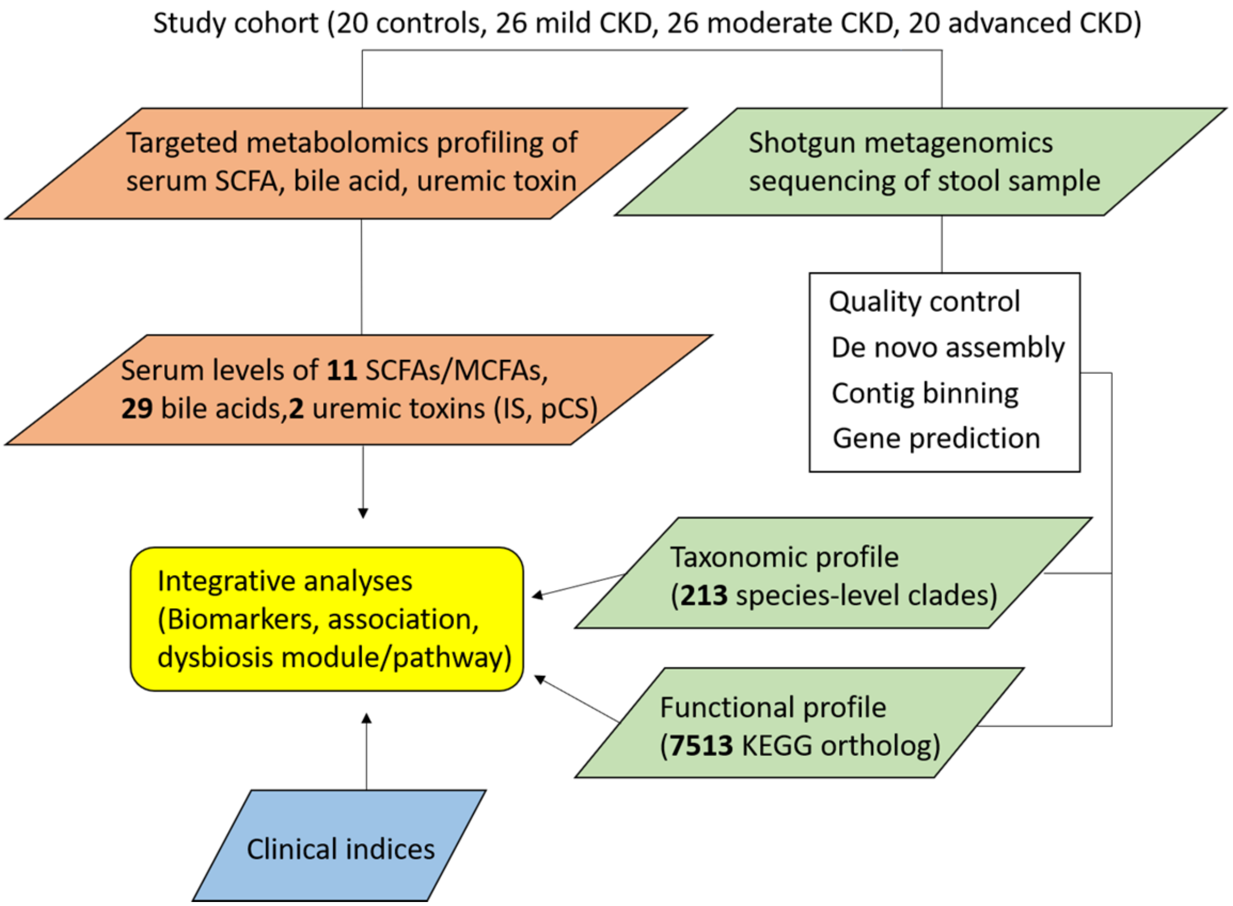

Supplemental figure 1. Overview of the study. Stool DNA samples from 92 subjects were used to obtain deep shotgun sequencing data, from which functional and species-level taxonomic profiles were generated. In addition, serum samples were used to conduct targeted metabolomics analysis to generate metabolite profiles. Samples from 92 subjects (20 non-CKD controls, 26 mild CKD, 26 moderate CKD, 20 advanced CKD) were available for both the sequencing and metabolomics data analyses. KEGG, Kyoto Encyclopedia of Genes and Genomes; SCFA, short-chain fatty acid; MCFA, medium-chain fatty acid; IS, indoxyl sulphate; pCS, p-cresyl sulphate.

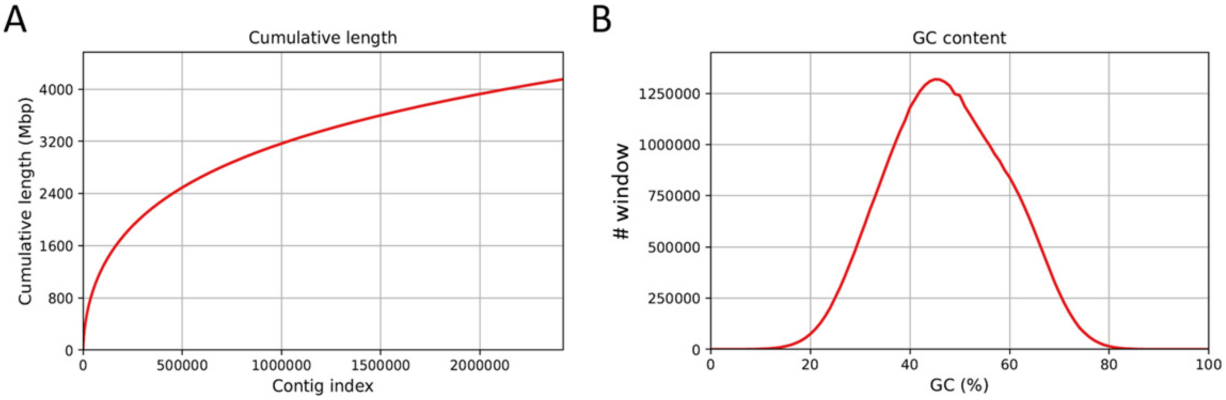

Supplemental figure 2. **(A)** Cumulative length of assembled contigs **(B)** Distribution of GC content of assembled contigs.

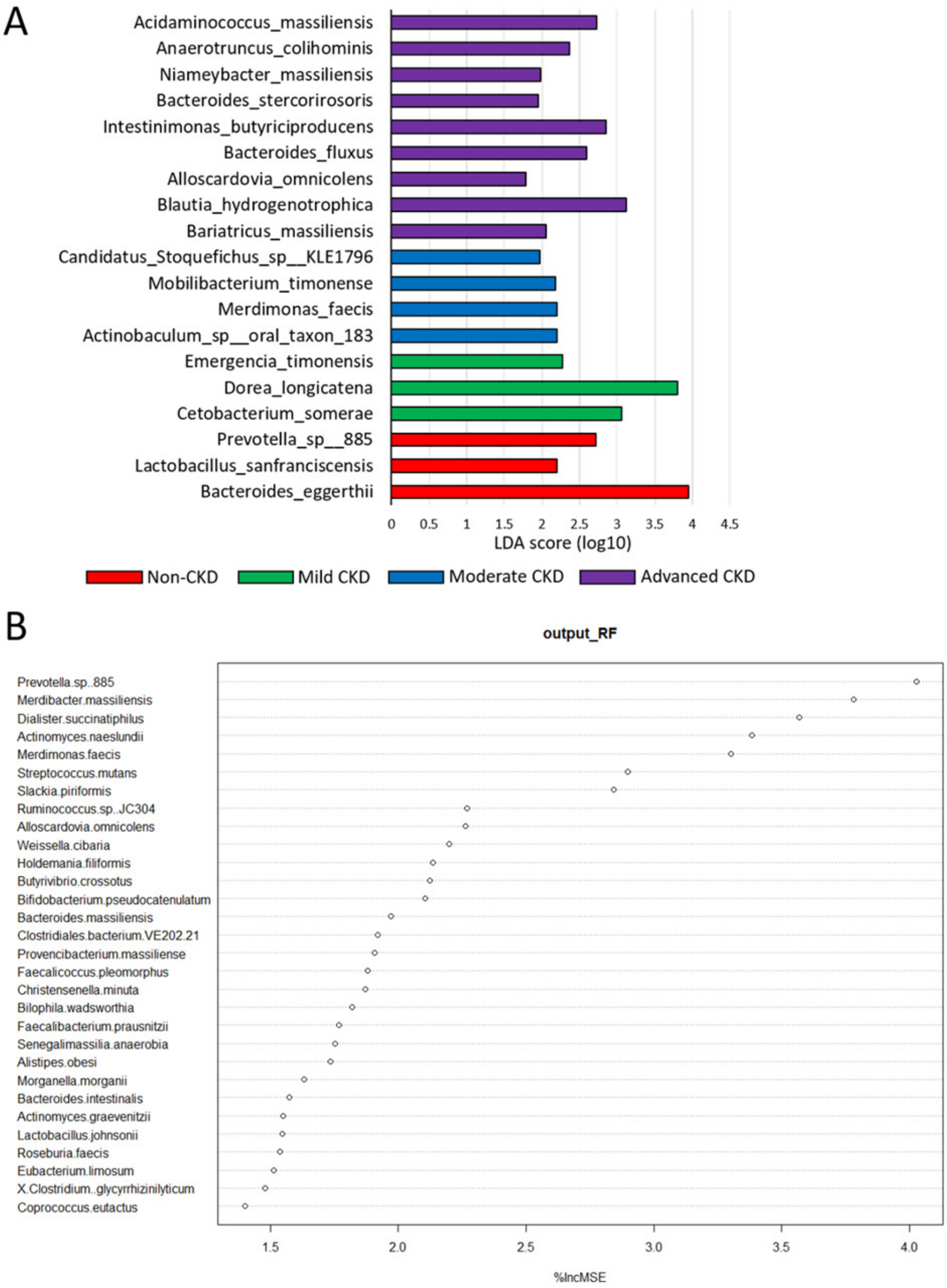

Supplemental figure 3. **Determination of bacterial biomarkers specific for each CKD stage or most discriminatory against the glomerular filtration rate. (A)** Gut microbes that best characterize each CKD group were identified by using linear discriminant analysis of effect size (LEfSe) on species-level abundance tables. **(B)** Species that are most discriminatory against renal dysfunction (glomerular filtration rate) were ranked in descending order of their importance to the accuracy of the model determined by applying Random Forests analysis.

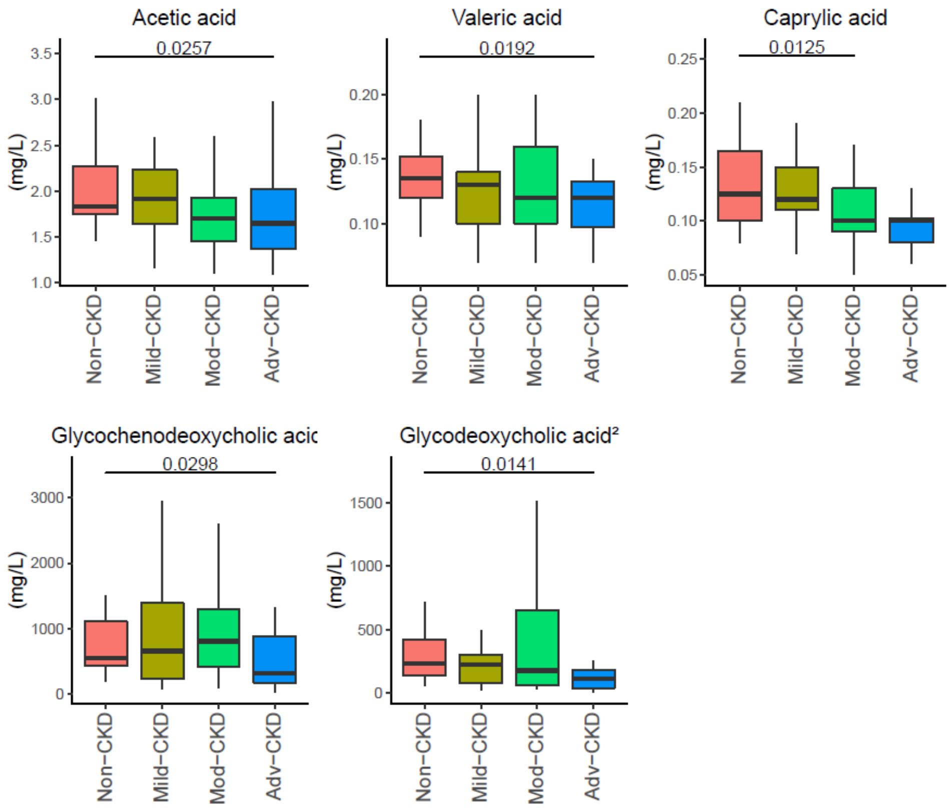

Supplemental figure 4. Comparison of circulating metabolic signatures across CKD groups.

Levels of metabolites among different groups were analyzed by Wilcoxon rank sum test.

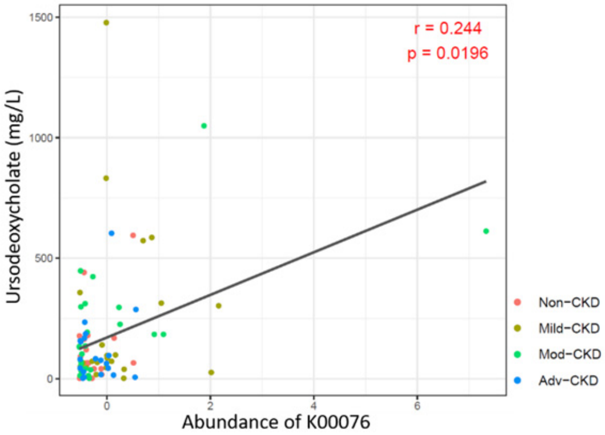

Supplemental figure 5. The correlation of ursodeoxycholic acid (Spearman's correlation, r = 0.244, *P* = 0.0196) with the abundance of K00076.

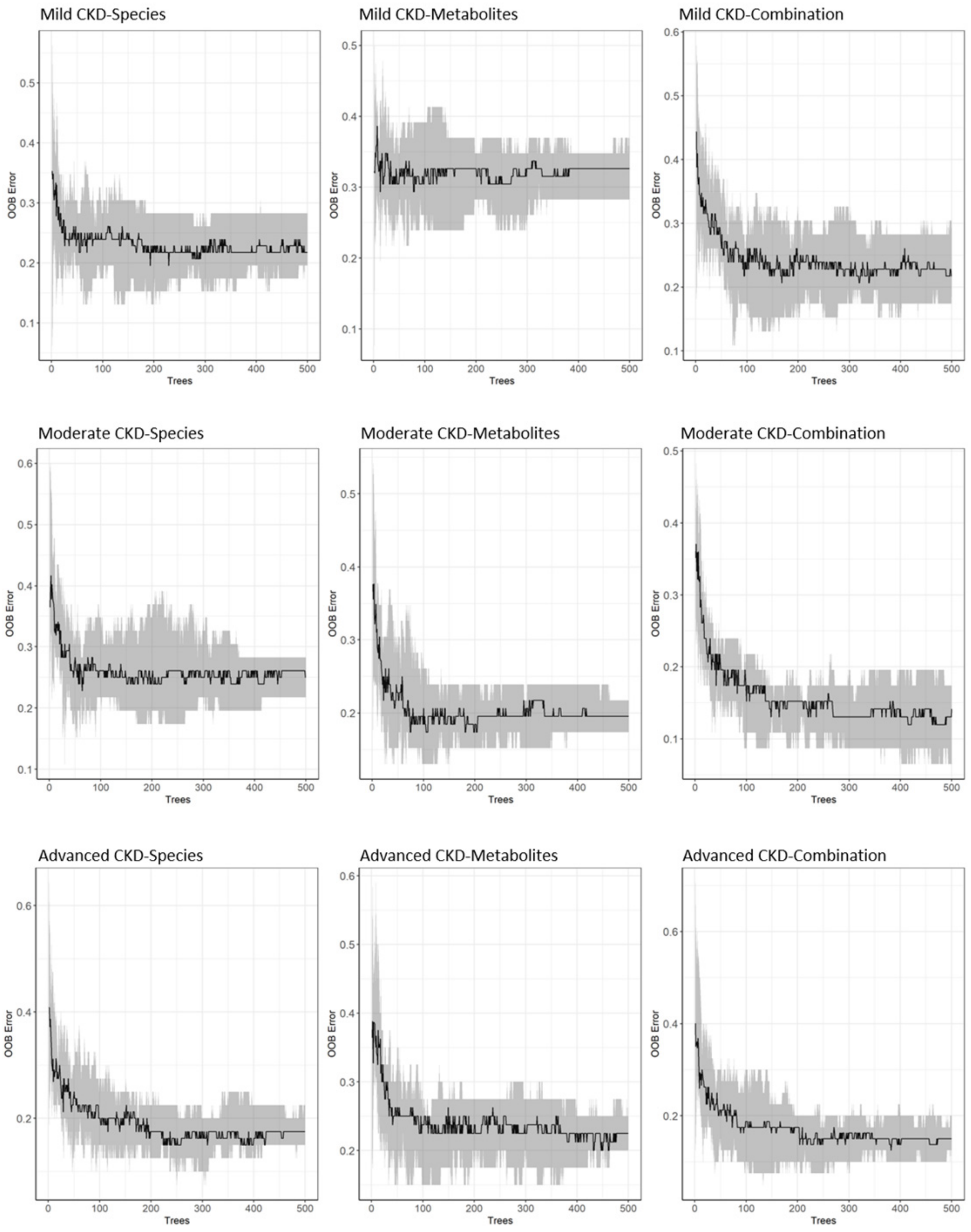

Supplemental figure 6. The estimated prediction error rate (out-of-bag error, OOB error) for biomarker changes with the size of the forest (the number of trees). The black lines represent the median of OOB error, and gray bands represent the range of minimum and maximum OOB error.

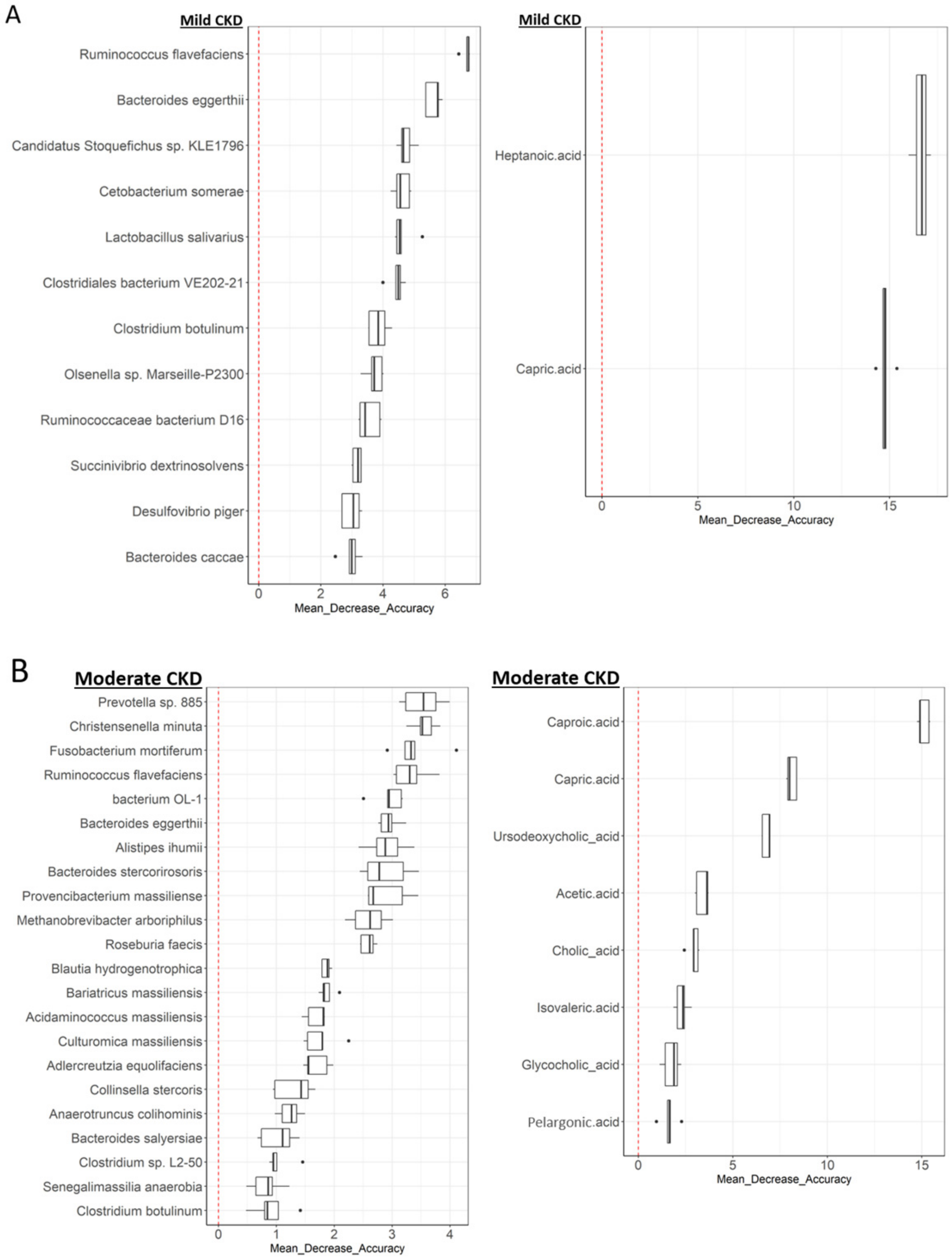
A
Mild CKD
Ruminococcus flavefaciens
Bacteroides eggerthii
Candidatus Stoquefichus sp. KLE1796
Cetobacterium somerae
Lactobacillus salivarius
Clostridiales bacterium VE202-21
Clostridium botulinum
Olsenella sp. Marseille-P2300
Ruminococcaceae bacterium D16
Succinivibrio dextrinosolvens
Desulfovibrio piger
Bacteroides caccae
0
2
4
6
Mean_Decrease_Accuracy
Mild CKD
Heptanoic.acid
Capric.acid
0
5
10
15
Mean_Decrease_Accuracy
B
Moderate CKD
Prevotella sp. 885
Christensenella minuta
Fusobacterium mortiferum
Ruminococcus flavefaciens
bacterium OL-1
Bacteroides eggerthii
Alistipes ihumii
Bacteroides stercorirosoris
Provencibacterium massiliense
Methanobrevibacter arboriphilus
Roseburia faecis
Blautia hydrogenotrophica
Bariatricus massiliensis
Acidaminococcus massiliensis
Culturomica massiliensis
Adlercreutzia equolifaciens
Collinsella stercoris
Anaerotruncus colihominis
Bacteroides salyersiae
Clostridium sp. L2-50
Senegalimassilia anaerobia
Clostridium botulinum
0
1
2
3
4
Mean_Decrease_Accuracy
Moderate CKD
Caproic.acid
Capric.acid
Ursodeoxycholic_acid
Acetic.acid
Cholic_acid
Isovaleric.acid
Glycocholic_acid
Pelargonic.acid
0
5
10
15
Mean_Decrease_Accuracy

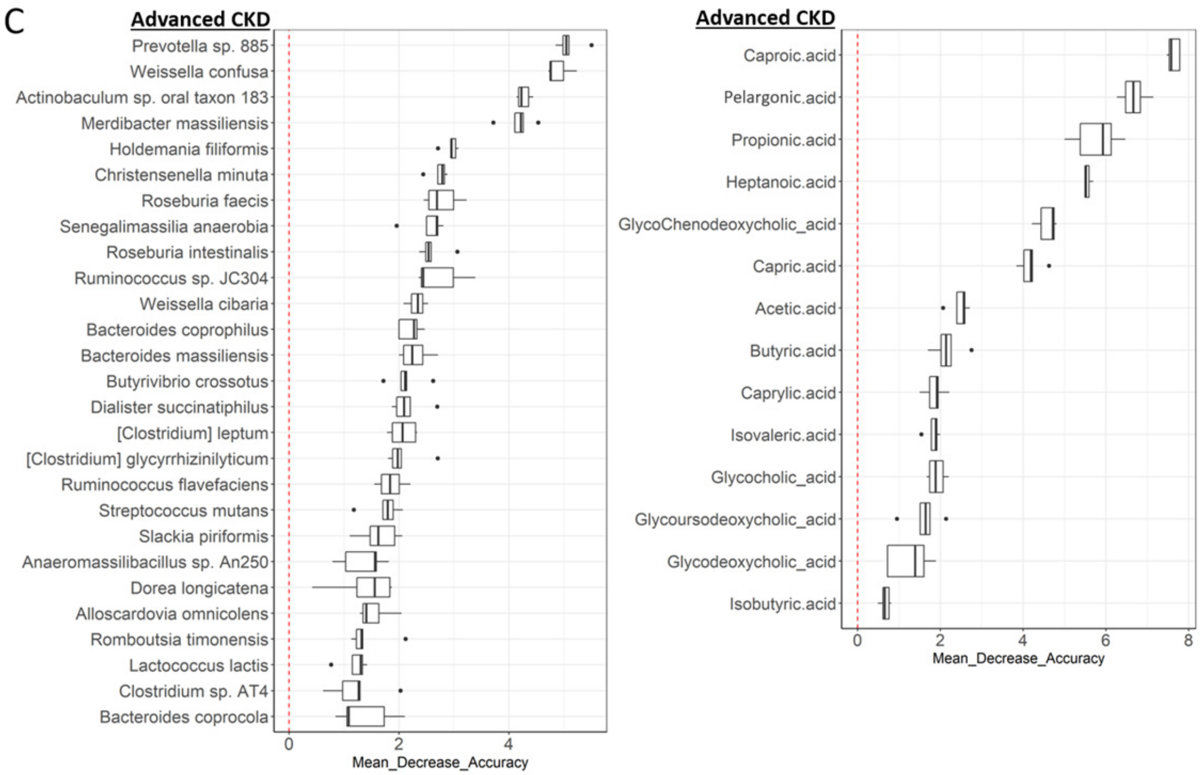

Supplemental figure 7. Metagenomic (left) and metabolomic (right) markers for detecting patients with mild **(A)**, moderate **(B)**, and advanced CKD **(C)** and early-stage CKD identified from Random Forests classifiers based on species-level taxonomic or metabolomic profiles. Markers are ranked in descending order of their importance to the accuracy of the model. The boxes represent 25th–75th percentiles, and black lines indicate the median.

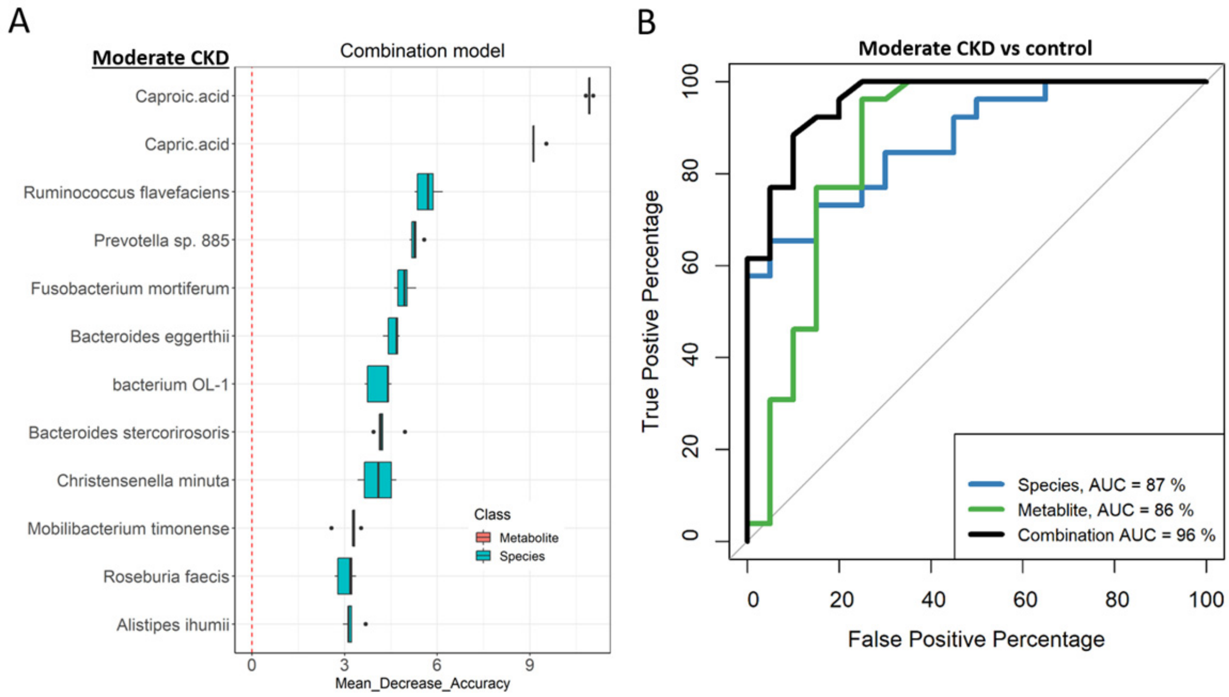

Supplemental figure 8. (A) Metagenomic and metabolomic markers for detecting patients with moderate CKD (n=26) from the controls (n=20) identified from Random Forests classifiers based on the combination of dual-omics markers. Markers are ranked in descending order of their importance to the accuracy of the model. The boxes represent 25th–75th percentiles, and black lines indicate the median. **(B)** ROC curves depict trade-offs between true and false positive rates for detecting patients with moderate CKD as classification stringency varies. AUC, the total area under the ROC curve.

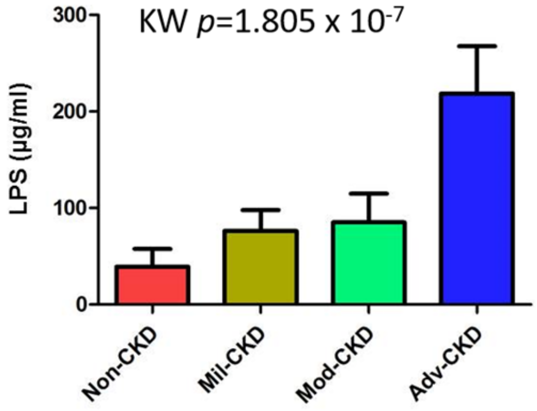

Supplemental figure 9. Comparison of circulating lipopolysaccharide (LPS) levels across CKD groups. Significant differences in serum levels of LPS among different groups were analyzed by Kruskal-Wallis test with a p value of 1.805×10^{-7}. Post-hoc P values of Dunn's test of multiple comparisons are 0.0017, 0.0009 and <0.0001 for the comparison of Non-CKD v.s. Mil-CKD, Non-CKD v.s. Mod-CKD and Non-CKD v.s. Adv-CKD, respectively.

Supplemental table 1. List of bile acids examined in this study.

no	Bile acid	abbv.	CAS	molecular formula
**1**	Dehydrolithocholic acid	DHLCA	1553-56-6	C24H38O3
**2**	Allolithocholic acid	alloLCA	2276-93-9	C24H40O3
**3**	Isolithocholic acid	isoLCA	1534-35-6	C24H40O3
**4**	Lithocholic acid	LCA	434-13-9	C24H40O3
**5**	23-Nordeoxycholic acid	23norDCA	53608-86-9	C23H38O4
**6**	7-Ketolithocholic acid	7-ketoLCA	4651-67-6	C24H38O4
**7**	12-Ketolithocholic acid	12-ketoLCA	5130-29-0	C24H38O4
**8**	Apocholic acid	apoCA	641-81-6	C24H38O4
**9**	Ursodeoxycholic acid	UDCA	128-13-2	C24H40O4
**10**	Hyodeoxycholic acid	HDCA	83-49-8	C24H40O4
**11**	Chenodeoxycholic acid	CDCA	474-25-9	C24H40O4
**12**	Deoxycholic acid	DCA	83-44-3	C24H40O4
**13**	Isodeoxycholic acid	isoDCA	566-17-6	C24H40O4
**14**	Dehydrocholic acid	DHCA	81-23-2	C24H34O5
**15**	7,12-Diketolithocholic acid	7,12-diketoLCA	517-33-9	C24H36O5
**16**	6,7-Diketolithocholic acid	6,7-diketoLCA	-	C24H36O5
**17**	7-Ketodeoxycholic acid	7-DHCA	911-40-0	C24H38O5
**18**	12-Dehydrocholic acid	12-DHCA	204023	C24H38O5
**19**	3-Dehydrocholic acid	3-DHCA	2304-89-4	C24H38O5
**20**	Ursocholic acid	UCA	2955-27-3	C24H40O5
**21**	α-Muricholic acid	α-MCA	2393-58-0	C24H40O5
**22**	β-Muricholic acid	β-MCA	2393-59-1	C24H40O5
**23**	λ-Muricholic acid	λ-MCA	547-75-1	C24H40O5
**24**	Allocholic acid	ACA	2464-18-8	C24H40O5
**25**	Cholic acid	CA	81-25-4	C24H40O5
**26**	Glycolithocholic acid	GLCA	24404-83-9	C26H43NO4
**27**	Glycoursodeoxycholic acid	GUDCA	64480-66-6	C26H43NO5
**28**	Glycohyodeoxycholic acid	GHDCA	38411-84-6	C26H43NO5
**29**	Glycochenodeoxycholic acid	GCDCA	16564-43-5	C26H43NO5
**30**	Glycodeoxycholic acid	GDCA	16409-34-0	C26H43NO5
**31**	Glycodehydrocholic acid	GDHCA	3415-45-0	C26H37NO6
**32**	Glyco-λ-muricholic acid	GλMCA	-	C26H43NO6
**33**	Glycocholic acid	GCA	475-31-0	C26H43NO6
**34**	Taurolithocholic acid	TLCA	6042-32-6	C26H45NO5S
**35**	Tauroursodeoxycholic acid	TUDCA	14605-22-2	C26H45NO6S
**36**	Taurohyodeoxycholic acid	THDCA	110026-03-4	C26H45NO6S
**37**	Taurochenodeoxycholic acid	TCDCA	516-35-8	C26H45NO6S

**38**	Taurodeoxycholic acid	TDCA	1180-95-6	C26H45NO6S
**39**	Tauro α-Muricholic acid	T-α-MCA	25696-60-0	C26H45NO7S
**40**	Tauro β-Muricholic acid	T-β-MCA	-	C26H45NO7S
**41**	Taurocholic acid	TCA	81-24-3	C26H45NO7S

Supplemental table 2. Time table-UHPLC-MS

	**Time**	**A (H2O)**	**B (ACN)**	**Flow**
1	0.0 min	75.0%	25.0%	0.40 mL/min
2	5.0 min	74.2%	25.8%	0.40 mL/min
3	5.5 min	71.5%	28.5%	0.40 mL/min
4	10.0 min	71.0%	29.0%	0.40 mL/min
5	12.0 min	64.0%	36.0%	0.40 mL/min
6	26.0 min	32.5%	67.5%	0.40 mL/min
7	26.2 min	1.0%	99.0%	0.40 mL/min
8	28.2 min	1.0%	99.0%	0.40 mL/min
9	28.4 min	75.0%	25.0%	0.40 mL/min
10	32.0 min	75.0%	25.0%	0.40 mL/min

Supplemental table 3. Statistics of assembled contigs

# contigs (>= 0 bp)	4685777
# contigs (>= 1000 bp)	1037907
# contigs (>= 5000 bp)	129084
# contigs (>= 10000 bp)	43346
# contigs (>= 25000 bp)	7483
# contigs (>= 50000 bp)	1605
Total length (>= 0 bp)	4975626734
Total length (>= 1000 bp)	3205419465
Total length (>= 5000 bp)	1435890325
Total length (>= 10000 bp)	849684732
Total length (>= 25000 bp)	326479799
Total length (>= 50000 bp)	130480093
# contigs	2420440
Largest contig	816575
Total length	4153936351
GC (%)	47.45
N50	2650
N75	1075
L50	309392
L75	951083
# N's per 100 kbp	0.00

All statistics are based on contigs of size ≥500 bp unless otherwise indicated.

Supplemental table 4. Annotation results of phylogenetic bins.

	Kingdom	Phylum	Class	Order	Family	Genus	Species
**Annotated bin#**	2	25	6	175	144	195	357
**Unique taxa#**	1	8	14	20	39	102	213
